# New approaches in the diagnosis and treatment of latent tuberculosis infection

**DOI:** 10.1186/1465-9921-11-169

**Published:** 2010-12-03

**Authors:** Suhail Ahmad

**Affiliations:** 1 Department of Microbiology, Faculty of Medicine, Kuwait University, Kuwait

## Abstract

With nearly 9 million new active disease cases and 2 million deaths occurring worldwide every year, tuberculosis continues to remain a major public health problem. Exposure to *Mycobacterium tuberculosis *leads to active disease in only ~10% people. An effective immune response in remaining individuals stops *M. tuberculosis *multiplication. However, the pathogen is completely eradicated in ~10% people while others only succeed in containment of infection as some bacilli escape killing and remain in non-replicating (dormant) state (latent tuberculosis infection) in old lesions. The dormant bacilli can resuscitate and cause active disease if a disruption of immune response occurs. Nearly one-third of world population is latently infected with *M. tuberculosis *and 5%-10% of infected individuals will develop active disease during their life time. However, the risk of developing active disease is greatly increased (5%-15% every year and ~50% over lifetime) by human immunodeficiency virus-coinfection. While active transmission is a significant contributor of active disease cases in high tuberculosis burden countries, most active disease cases in low tuberculosis incidence countries arise from this pool of latently infected individuals. A positive tuberculin skin test or a more recent and specific interferon-gamma release assay in a person without overt signs of active disease indicates latent tuberculosis infection. Two commercial interferon-gamma release assays, QFT-G-IT and T-SPOT.TB have been developed. The standard treatment for latent tuberculosis infection is daily therapy with isoniazid for nine months. Other options include therapy with rifampicin for 4 months or isoniazid + rifampicin for 3 months or rifampicin + pyrazinamide for 2 months or isoniazid + rifapentine for 3 months. Identification of latently infected individuals and their treatment has lowered tuberculosis incidence in rich, advanced countries. Similar approaches also hold great promise for other countries with low-intermediate rates of tuberculosis incidence.

## Introduction

Tuberculosis (TB) is a formidable public health challenge as it contributes considerably to illness and death around the world. The most common causative agent of TB in humans, *Mycobacterium tuberculosis*, is a member of the *M. tuberculosis *complex (MTBC) which includes six other closely related species: *M. bovis, M. africanum, M. microti, M. pinnipedii, M. caprae *and *M. canettii*. All MTBC members are obligate pathogens and cause TB; however, they exhibit distinct phenotypic properties and host range. Genetically, MTBC members are closely related, the genome of *M. tuberculosis *shows >99.9% similarity with *M. bovis*, the species that primarily infects cattle but can also cause TB in other mammals including man [1,2]. The current TB epidemic is being sustained by two important factors; the human immunodeficiency virus (HIV) infection and its association with active TB disease and increasing resistance of *M. tuberculosis *strains to the most effective (first-line) anti-TB drugs [3-5]. Other contributing factors include population expansion, poor case detection and cure rates in impoverished countries, wars, famine, diabetes mellitus and social decay and homelessness [6,7].

According to recent estimates, 9.4 million new active disease cases corresponding to an estimated incidence of 139 per 100,000 population occurred throughout the world in 2008 [3,4]. Only 5.7 million of 9.4 million cases of TB (new cases and relapse cases) were notified to national tuberculosis programs of various countries while the rest were based on assessments of effectiveness of surveillance systems. The highest number of TB cases occurred in Asia (55%) followed by Africa (30%). The highest incidence rate (351 per 100,000 population) was recorded for the African region, mainly due to high prevalence of HIV infection. An estimated 1.4 million (15%) of incident TB patients were coinfected with HIV in 2008. Globally, the total prevalent TB cases in 2008 were 11.1 million corresponding to 164 cases per 100 000 population that resulted in 1.8 million deaths (including 0.5 million TB patients coinfected with HIV) [3,4]. Nearly 440 000 cases of multidrug-resistant TB (MDR-TB, defined as infection with *M. tuberculosis *strains resistant at least to the two most important first-line drugs, rifampicin and isoniazid) occurred in 2008 [5]. By 2009, extensively drug-resistant TB (XDR-TB; defined as MDR-TB strains additionally resistant to a fluoroquinolone and a second-line anti-TB injectable agent such as kanamycin, amikacin, or capreomycin) has been found in 58 countries [5]. While MDR-TB is difficult and expensive to treat, XDR-TB is virtually an untreatable disease in most of the developing countries [8].

## Establishment and persistence of latent *M. tuberculosis *infection

**Figure 1:**
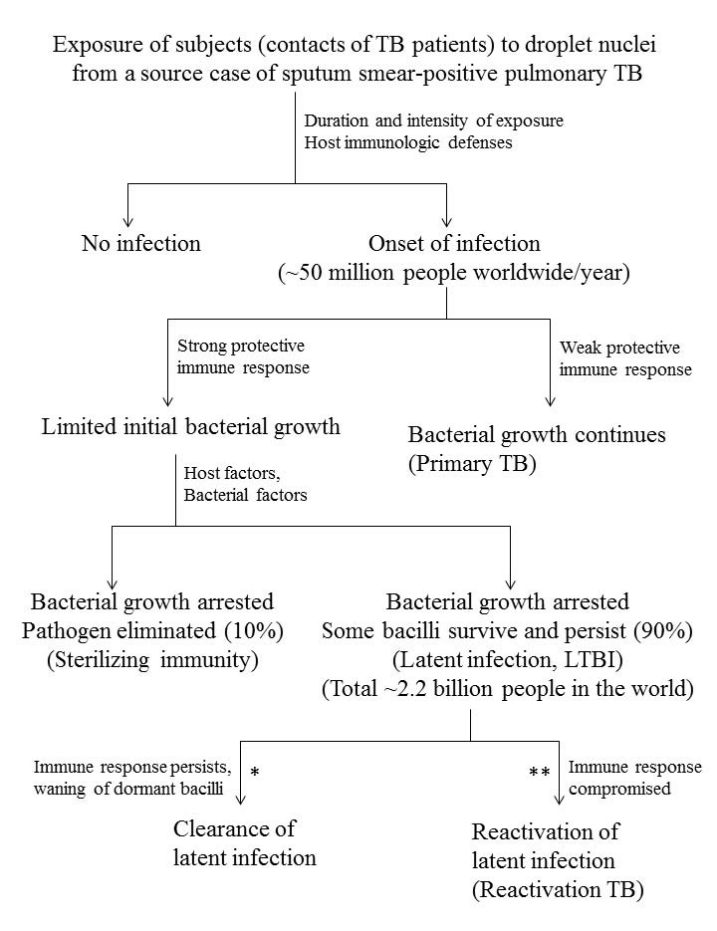
**Natural progression of events and outcome in an immunocompetent individual following exposure of human subjects (contacts of TB patients) to droplet nuclei containing *M. tuberculosis *expectorated by a source case of sputum smear-positive pulmonary TB**. Every year, ~50 million people worldwide are infected with *M. tuberculosis*. Complete elimination of tubercle bacilli is achieved in ~10% individuals only while in ~90% of infected individuals, bacterial growth is stopped but some bacilli survive and persist leading to latent *M. tuberculosis *infection (LTBI). The waning of dormant bacilli in persons with LTBI can be accelerated by therapy with isoniazid for 9 months (denoted by *). The vaccines currently in clinical trials are designed to prevent or delay the reactivation of latent infection in persons with LTBI (denoted by **).

Tuberculosis is a communicable disease and infection is initiated by inhalation of droplet nuclei (1-5 μm in diameter particles) containing *M. tuberculosis*, expectorated by patients with active pulmonary or laryngeal TB, typically when the patient coughs. Active transmission occurs more frequently in small households and crowded places in countries with a high incidence of TB and the risk of infection is dependant on several factors such as the infectiousness of the source case, the closeness of contact, the bacillary load inhaled and the host's immune status (Figure 1) [9-11]. Molecular epidemiological studies have shown that there are distinct differences in the disease presentation and population demographics in low TB incidence and high TB incidence countries. In several African and Asian countries, the vast majority of mycobacterial infections are caused by *M. tuberculosis *and incidence rates are highest among young adults, with most cases resulting from recent episodes of infection or reinfection [12-14]. On the contrary, in low TB incidence countries of Western Europe and North America, a higher proportion of active TB cases occur in older patients or among immigrants from high TB incidence countries [12]. Pulmonary TB accounts for >85% of active TB cases in high TB incidence countries while extrapulmonary TB is more common in low TB incidence countries, particularly among HIV infected individuals and immigrants originating from TB endemic countries [15,16].

The inhaled droplet nuclei avoid the defenses of the bronchi due to their small size and penetrate into the terminal alveoli of the lungs where they are engulfed by phagocytic antigen-presenting cells including alveolar macrophages, lung macrophages and dendritic cells. In the lungs, *M. tuberculosis *can also infect non-phagocytic cells in the alveolar space such as endothelial cells, M cells and type 1 and type 2 epithelial cells [17-20]. In the initial phase of infection, *M. tuberculosis *internalized by macrophages and dendritic cells replicates intracellularly and the bacteria-laden immune cells may cross the alveolar barrier to cause systemic dissemination [18,19]. The intracellular replication and simultaneous dissemination of the pathogen to the pulmonary lymph nodes and to various other extrapulmonary sites occurs prior to the development of the adaptive immune responses [21,22].

The entry of *M. tuberculosis *in phagocytic immune cells in the alveolar space begins with recognition of pathogen-associated molecular patterns by specific pathogen recognition receptors that initiate a coordinated innate immune response by the host [23]. The *M. tuberculosis *components are recognized by host receptors that include toll-like receptors (TLRs), nucleotide-binding oligomerization domain (NOD)-like receptors (NLRs), and C-type lectins [24-26]. The C-type lectins include mannose receptor (MR), the dendritic cell-specific intercellular adhesion molecule grabbing nonintegrin (DC-SIGN), macrophage inducible C-type lectin (Mincle) and dendritic cell-associated C-type lectin-1 (Dectin-1) [24,27]. The TLR signaling is the main arm of the innate immune response and *M. tuberculosis *internalized through different receptors may also have different fate [28-30].

The *M. tuberculosis *cell envelope is composed of a cell wall that is covered with a thick waxy mixture of lipids, polysaccharides and mycolic acids. The most important *M. tuberculosis *cell surface ligands that interact with TLRs and other receptors include the 19 and 27 kDa lipoproteins, 38 kDa glycolipoprotein, glycolipids (such as phosphatidylinositol mannoside, PIM; lipomannan, LM; lipoarabinomannan, LAM; and mannose-capped lipoarabinomannan, Man-LAM) and trehalose dimycolate (TDM) (Table 1) [26,28,30,31]. Other ligands may include surface exposed proteins such as LprA and LprG lipoproteins and mammalian cell entry (Mce) proteins encoded by the *mce1 *and *mce3 *operons [32-36]. Typically, signals generated through TLR and Mincle promote proinflammatory immune responses while preferential recruitment of DC-SIGN induces suppression and/or exhaustion of immune responses [25,27,30,37]. The glycolipids (such as PIM, LM and, LAM) and lipoproteins (such as 19 kDa lipoprotein, LpqH) that are exposed on *M. tuberculosis *cell surface [38] are mainly recognized by TLR2 (Table 1) [24,26,30].

**Table 1:** Important M. tuberculosis ligands, main receptors on phagocytic immune cells and immune cell processes affected that promote persistence of the pathogen and establishment of latent tuberculosis infection in humans

*M. tuberculosis *ligand^a^	Host cell receptor^b^	Immune cell process affected	Reference(s)
19 kDa Lipoprotein (LpqH)	TLR2	MHC class II expression/antigen presentation	28,30,90

19 kDa Lipoprotein (LpqH)	TLR2	Phagosomal processing by MHC class I pathway	89,96

Lipoprotein LprA	TLR2	MHC class II expression/antigen presentation	33

Lipoprotein LprG	TLR2	MHC class II expression/antigen presentation	32

Phosphatidyinositol mannoside (PIM)	TLR2	Modulation of macrophage signaling pathways	26,51

Lipomannan (LM)	TLR2, MR	Modulation of macrophage signaling pathways	26,51

Lipoarabinomannan (LAM)	TLR2	Modulation of macrophage signaling pathways	26,51

Mannose-capped LAM	MR, DC-SIGN	Phagolysosome maturation	91,92

Mannose-capped LAM	MR, DC-SIGN	MHC class II expression/antigen presentation	51,91.96

Mannose-capped LAM	MR, DC-SIGN	IL-12 secretion of dentritic cells/macrophages	88

Mannose-capped LAM	MR, DC-SIGN	Apoptosis of macrophages	91,112

Trehalose dimycolate (cord factor)	TLR2, Mincle	Phagolysosome biogenesis	27,93,95

Trehalose dimycolate (cord factor)	TLR2, Mincle	MHC class II expression/antigen presentation	27,94,95

^a^Mannose-capped LAM, Mannose-capped lipoarabinomannan

^b^TLR2, Toll-like receptor 2; MR, mannose receptor; DC-SIGN, dendritic cell-specific intercellular adhesion molecule grabbing nonintegrin; Mincle, macrophage inducible C-type lectin

The interaction of *M. tuberculosis *ligand(s) with TLRs initiates an intracellular signaling cascade that culminates in a proinflammatory response (beneficial to the host), however, the bacterium has also evolved strategies that can trigger signals that dampen the innate immune response (beneficial to the pathogen). The proinflammatory process results in activation of nuclear transcription factor (NF)-κB and production of proinflammatory cytokines, chemokines and nitric oxide through either myeloid differentiation primary response protein 88 (MyD88)-dependant or MyD88-independent pathway [24,30,39-41]. A brief outline of the immune response of the host is described here. Several excellent review articles are available for a more detailed description [25,42-45].

In addition to macrophages and dendritic cells, a wide range of other immune components are also involved in an effective immune response against *M. tuberculosis *and include, αβ-T cells (both CD4^+ ^and CD8^+^), CD1 restricted T cells, γδ-T cells and cytotoxic T cells as well as the cytokines produced by these immune cells [25,45-47]. The most important among these are CD4^+ ^T cells and the cytokine interferon (IFN)-γ.

The two major defense mechanisms of macrophages include the fusion of the phagosomes containing *M. tuberculosis *with lysosomes (phagolysosome) that is bactericidal and generation of nitric oxide and other reactive nitrogen intermediates (RNI) which exert toxic effects on the bacilli [43,45,48-51]. The *M. tuberculosis *containing phagosomes mature through a series of fusion and fission events with several endocytic vesicles that culminate in a phagolysosome. The fusion-fission events remodel the phagosomal membrane. The Ca^+2 ^signaling cascade and recruitment of vacuolar-proton transporting ATPase (vH^+^-ATPase) cause lowering of internal pH that allows lysosome-derived acid hydrolases to function efficiently for their microbicidal effect [52-54]. Another mycobactericidal mechanism of macrophages includes lysosomal killing of *M. tuberculosis *mediated by ubiquitin-derived peptides [55]. The ubiquitination destroys tubercle bacilli by autophagy as a ubiquitin-derived peptide impairs the membrane integrity of *M. tuberculosis *that allows nitric oxide to kill more efficiently. The apoptosis of infected macrophages participates in host defense against infection as apoptotic vesicles containing mycobacterial antigens are taken up by dendritic cells for CD8^+ ^T cell activation by phagosome-enclosed antigens [25,56,57].

Mycobacterial antigens in macrophages or dendritic cells are picked up by the MHC class II molecules and presented to CD4^+ ^T cells [28,32,43]. The phagosomal membrane is also equipped with the MHC class I processing machinery [58,59]. Also, CD1 proteins present glycolipids, lipids, and lipopeptides of lipid-rich *M. tuberculosis *to T cells [56,60,61]. Furthermore, the vesicles formed due to apoptosis of *M. tuberculosis*-infected macrophages are taken up by dendritic cells and presented to the T cells through the MHC class I and CD1 molecules [56,61].

Immediately after entry of *M. tuberculosis*, alveolar macrophages produce inflammatory cytokines and chemokines that serve as a signal for infection. The monocytes, neutrophils and lymphocytes migrate to the focal site of infection but they are unable to kill the bacteria efficiently. During this time, the bacilli resist the bactericidal mechanisms of the macrophage (phagolysosome) by preventing phagosome-lysosome fusion, multiply in the phagosome and eventually escape from phagosome/phagolysosome and cause macrophage necrosis [44,51]. The escape of *M. tuberculosis *from phagosome/phagolysosome is aided by the 6-kDa early secreted antigenic target (ESAT-6) protein and ESX-1 protein secretion system encoded by the region of difference 1 (RD1), a genomic segment that is present in all virulent *M. tuberculosis *and *M. bovis *strains but is absent in the vaccine strain *M. bovis *BCG [1,2,62-68]. The ESAT-6 protein associates with liposomes containing dimyristoylphosphatidylcholine and cholesterol and causes destabilization and lysis of liposomes [67]. It has also been shown that ESAT-6, released during acidification of phagosome from ESAT-6:10 kDa-culture filtrate protein (CFP-10) complex (secreted by live *M. tuberculosis *through ESX-1 secretion system), inserts itself into lipid bilayer and causes lysis of phagosome and escape of tubercle bacilli [69]. The ESAT-6 also induces apoptosis of macrophages via the caspase-dependent pathway and cytolysis of type 1 and type 2 alveolar epithelial cells and helps in the dissemination of *M. tuberculosis *[20,70].

The released bacilli multiply extracellularly, are phagocytosed by another macrophage that also fails to control the growth of *M. tuberculosis *and likewise is destroyed [42,43,51,71,72]. This progression of events continues unabated (in persons with a weak immune response) leading to active TB disease in ~10% of individuals (Primary TB) (Figure 1). In vast majority of the infected individuals, however, an effective cell-mediated immune response develops 2-8 weeks after infection as dendritic cells with engulfed bacilli mature, migrate to the regional lymph node and prime T cells (both CD4^+ ^and CD8^+^) against *M. tuberculosis *antigens [25,45,73]. The specific immune response produces primed T cells which migrate back to the focus of infection, guided by the chemokines produced by infected cells. The accumulation of macrophages, T cells and other host cells (dendritic cells, fibroblasts, endothelial cells and stromal cells) leads to the formation of granuloma at the site of infection [74,75]. The CD4^+ ^T cells producing IFN-γ recognize infected macrophages presenting antigens from *M. tuberculosis *and kill them [43,45,76].

The early stages of granuloma formation appear to benefit *M. tuberculosis *as ESAT-6 promotes accumulation of macrophages of different activation and maturation stages at the site of infection in which the tubercle bacilli multiply unabated and infected macrophages may also transport the pathogen to other sites in the body [22,77]. The eventual formation of solid granuloma due to an effective immune response walls off tubercle bacilli from the rest of the lung tissue, limits bacterial spread and provide microenvironment for interactions among macrophages and other immune cells and the cytokines. It is also apparent now that *M. tuberculosis *infected individuals show differences in the innate immune responses that lead to the formation of physiologically distinct granulomatous lesions. Some of these lesions eliminate all bacilli (sterilizing immunity) while others allow persistence of viable *M. tuberculosis *in the microenvironment [75,78]. Low-dose infection in primate models of human latent TB exhibit at least two types of tuberculous granuloma [79,80]. The classic caseous granuloma are composed of epithelial macrophages, neutrophils, and other immune cells surrounded by fibroblasts. *M. tuberculosis *resides inside macrophages in the central caseous necrotic region that is hypoxic [80,81]. The second type of granulomas (fibrotic lesions) are composed of mainly fibroblasts and contain very few macrophages, however, the exact location of viable *M. tuberculosis *in these lesions is not known [80].

With granuloma formation and an effective immune response, most tubercle bacilli are killed and disease progression is halted [42,45,75]. Although proinflammatory immune response is generally beneficial to the host, restricting this response is essential to avoid the risk of producing excessive inflammation that could damage host tissues. This is accomplished through a family of receptor tyrosine kinases that provide a negative feedback mechanism to both, TLR-mediated and cytokine-driven proinflammatory immune responses [82,83]. This defense mechanism of the host has been exploited by *M. tuberculosis *for its survival [84-87]. Several *M. tuberculosis *factors such as 19-kDa lipoprotein, glycolipids (particularly Man-LAM), trehalose dimycolate (cord factor) and several others (Table 1) can modulate antigen-processing pathways by MHC class I, MHC class II and CD1 molecules, phagolysosome biogenesis and other macrophage signaling pathways [26-28,30,32,33,88-95]. The suppression of these responses blunt the microbicidal functions of macrophages and other immune cells (such as reactive nitrogen intermediates) or prevent their proper maturation (phagolysosome) [24,26,30,45,51,96].

The inhibition of macrophage responses to *M. tuberculosis *results in a subset of infected macrophages that are unable to present *M. tuberculosis *antigens to CD4^+ ^T cells. This results in insufficient activation of effector T cells leading to evasion of immune surveillance and creation of niches where *M. tuberculosis *survives [45,51,96,97]. The hypoxia, nutrient deficiency, low pH and inhibition of respiration by nitric oxide in the microenvironment of the granuloma induce a dormancy program in *M. tuberculosis *[98,99]. These conditions transform surviving bacilli into a dormant stage with little or no metabolic and replicative activity, however, expression of DosR-regulated dormancy antigens continues [99-101]. It is also probable that *M. tuberculosis*, under these conditions, forms spore-like structures, typically seen with other mycobacteria in response to prolonged stationary phase or nutrient starvation, for its survival [102]. Decreased outer membrane permeability also protects *M. tuberculosis *from killing by ubiquitin-derived peptides [103]. Thus, some non-replicating (resistant) bacilli avoid elimination by the immune system and persist. This latent tuberculosis infection (LTBI) in a person without overt signs of the disease is indicated by the delayed-type hypersensitivity (DTH) response to purified protein derivative (PPD) prepared from culture filtrates of *M. tuberculosis *(tuberculin skin test) [9,104]. The dormant bacilli can inhabit the granuloma during the lifetime of the host but are able to resume their growth if (or when) the immune response is compromised (reactivation TB) (Figure 1). The World Health Organization (WHO) has estimated that one-third of the total world population is latently infected with *M. tuberculosis *and 5%-10% of the infected individuals will develop active TB disease during their life time [104]. However, the risk of developing active disease is 5%-15% every year and lifetime risk is ~50% in HIV coinfected individuals [3,4,105].

Reactivation of latent infection requires *M. tuberculosis *to exit dormancy. The lytic transglycosylases known as resuscitation promoting factors and an endopeptidase (RipA) of *M. tuberculosis *have been recognized as vital components for revival from latency [106-108]. Although reactivation of latent infection can occur even decades after initial infection, a person is at greater risk of developing active TB disease during the first two years after infection with *M. tuberculosis *[9,109,110]. Several factors can trigger development of active disease from reactivation of remote infection, and typically involve the weakening of the immune system [111]. HIV infection is the most important risk factor for progression to active disease in adults as it causes depletion/functional abnormalities of CD4^+ ^and/or CD8^+ ^T-cells that are central for protection against active TB disease [3,4,6,105]. Likewise, *M. tuberculosis *infection accelerates the progression of asymptomatic HIV infection to acquired immunodeficiency syndrome (AIDS) and eventually to death. This is recognized in the current AIDS case definition as pulmonary or extrapulmonary TB in HIV-infected patient is sufficient for the diagnosis of AIDS. The reactivation TB can occur in any organ system, however, in immunocompetent individuals, it usually occurs in the upper lobes, where higher oxygen pressure supports good bacillary growth.

## New dynamic model of latent tuberculosis infection

The traditional model of LTBI as described in detail above begins with the entry of *M. tuberculosis *in antigen-presenting cells in lung alveoli and the pathogen accomplishes intracellular survival through several evasion strategies including neutralization of the phagosomal pH, antigen presentation by macrophages and dendritic cells that compromise CD4^+ ^T cell stimulation, apoptosis of infected macrophages and interference with autophagy [51,75,111,112]. The early stages of developing granuloma benefit the pathogen as it invades macrophages of different activation and maturation stages and thus, survives when the loose aggregates of phagocytes and polymorphonuclear granulocytes transform into a solid granuloma [75,77,111]. Although active disease is averted for the moment, latent infection ensues as the pathogen is not eliminated. The tubercle bacilli are resistant to immune attack as they are transformed into a dormant stage with very low or nil metabolic and replicative activity, however, a dormancy-related gene set called DosR regulon continues to be expressed during latent infection [99,101]. The exact physical and metabolic nature and location of persistent tubercle bacilli in the dormant state remains unknown. The bacilli can remain dormant for the entire life of the host without ever causing active disease or they may cause disease several years or even decades later [109,110]. Impaired immunity due to exhaustion or suppression of T cells results in resuscitation of *M. tuberculosis *from a dormant to a metabolically active stage leading to active TB disease (reactivation TB) [25,101]. However, the risk of developing reactivation TB disease is highest during the first two years after infection with *M. tuberculosis *[109,113]. Similarly, reactivation TB in immunocompetent individuals immigrating from TB endemic countries to low TB incidence countries also occurs usually within the first two years of their migration [6,9,113,114]. Based on these observations and some recent experimental data, a dynamic model of latent infection has been proposed recently in which endogenous reactivation as well as damage response occurs constantly in immunocompetent individuals [115].

The model suggests that during initial stages (developing granuloma) of infection, *M. tuberculosis *grow well inside phagosome and then escape from phagosome/phagolysosome and are released in extracellular milieu due to macrophage necrosis [69,70,116,117]. Some of the extracellular bacilli stop replicating due to hypoxic and acidic environment, nutrient limitation (conditions that mimic stationary bacterial cultures) and presence of bactericidal enzymes released from destruction of immune cells, even before an effective immune response is fully developed. With the development of an effective immune response, the actively growing bacilli are easily killed, however, the metabolically inactive, non-replicating (dormant) bacilli resist killing and may survive [116].

The model also assigns an important role to foamy macrophages that emerge during chronic inflammatory processes (such as TB) due to phagocytosis of cellular debris rich in fatty acids and cholesterol in the dissemination and/or waning of infection. The model suggests that as foamy macrophages phagocytose extracellular non-replicating lipid-rich *M. tuberculosis *along with other cellular debris, the bacilli are not killed due to their non-replicating, metabolically inert (dormant) state. At the same time, tubercle bacilli also do not grow in the intracellular environment as the macrophages are now activated [118-120]. As the foamy macrophages containing non-replicating bacilli drain from lung granuloma towards bronchial tree, they lodge *M. tuberculosis *into a different region of lung parenchyma due to aerosols generated by inspired air and the bacilli get another chance to begin the infection process at this new location [115,118,119,121]. In this infection-control of growth-reinfection process, bacilli getting lodged in the upper lobe may have the chance to cause cavitary lesion. This is due to higher oxygen pressure in upper lobes that can support rapid extracellular bacillary growth resulting in bacillary concentration that can not be controlled by the optimum immune response mounted by the host. The subsequent much stronger inflammatory response leads to tissue destruction, liquefaction and extracellular bacillary growth which amplifies the response further and causes cavitation [115,116].

The dynamic infection model, involving drainage and destruction of non-replicating bacilli in the stomach over a period of time, proposes slow clearance (waning) of latent infection in a sub-set of infected individuals who are not at risk of reinfection. A recent study carried out in Norway, a country with a low risk of active transmission of infection or reinfection, has shown that rates of reactivation TB, among patients previously exposed to *M. tuberculosis*, have progressively declined over the last several years [122]. Furthermore, the prevention of reinfection by bacilli resuscitated from dormancy by isoniazid, during infection-control of growth-reinfection cycles, also explains how therapy for only nine months with a single drug, effective only against actively dividing bacilli, is highly effective for a latent infection sustained by non-replicating bacilli that can presumably survive during the lifetime of the host [115].

## Diagnosis of latent *M. tuberculosis *infection

Despite the fact that control and management of TB in many low TB incidence countries is centered around the identification and subsequent treatment of individuals latently infected with *M. tuberculosis *(LTBI), actual identification of LTBI in human subjects is presently not feasible [123,124]. The current diagnostic tests (such as the tuberculin skin test or more recently developed T cell-based assays) are only designed to measure the adaptive immune response of the host exposed to *M. tuberculosis*, typically six to eight weeks after exposure to the bacilli [123-126].

The tuberculin skin test (TST) measures cell-mediated immunity in the form of a DTH response to a complex cocktail of >200 *M. tuberculosis *antigens, known as purified protein derivative (PPD) and the test result is usually read as induration (in mm) recorded 48 to 72 hours after intradermal injection of PPD [127]. The criteria for a positive TST vary considerably and depend on the inoculum and type of PPD preparation used in the test. In the United States, 5 tuberculin units (TUs) are generally used and the induration of ≥5 mm in HIV-seropositive or organ transplant recipient or in a person in contact with a known case of active TB is considered as positive [128]. However, in foreign-born persons originating from high TB incidence countries or persons at higher risk of exposure to *M. tuberculosis *(such as health care professionals), induration of ≥10 mm is regarded as positive TST [128]. In most European countries, 2 TUs are used and the induration of ≥10 mm in immunocompetent adults is considered as positive. In the United Kingdom, 10 TUs are used and the induration of 5-15 mm in BCG unvaccinated and ≥15 mm in BCG vaccinated immunocompetent adults is considered as positive [123-126]. Skin test reaction over 20 mm is usually due to active disease; however, a negative skin test in an active TB patient may also result from anergy or incorrect administration of the test or improper storage of the test reagents, thus compromising the sensitivity of the test [9,104,127,128]. Skin testing is most suitable for detecting *M. tuberculosis *infection in developing countries where >80% of the global TB cases occur, as it does not require extensive laboratory facilities and health care workers are already familiar with administering and reading skin tests. However, TST has several inherent problems as the antigens present in PPD are also present in the vaccine strain *M. bovis *BCG and several environmental mycobacteria. Hence, TST has lower specificity as the test can not differentiate between infection with *M. tuberculosis*, prior vaccination with *M. bovis *BCG or sensitization with environmental mycobacteria [9,104,127,129,130]. Furthermore, sensitivity of TST is limited in immunocompromised individuals due to anergy. These factors have compromised the sensitivity and specificity of tuberculin skin test for the diagnosis of LTBI.

Highly sensitive and more specific tests for the diagnosis of LTBI have been developed recently as a result of advances in genomics and immunology. The availability of complete genome sequences of *M. tuberculosis *and other *Mycobacterium *spp. and subtractive hybridization-based approaches identified RD1, a genomic region that is present in all *M. tuberculosis *and pathogenic *M. bovis *strains but is absent in all *M. bovis *BCG vaccine strains and most of the environmental mycobacteria of clinical relevance [13,64,65]. Two of the RD1 encoded proteins, ESAT-6 and CFP-10 are strong T cell antigens [62,63]. Early studies in animals showed that DTH skin responses to ESAT-6 and CFP-10 discriminated between animals infected with *M. tuberculosis *from those sensitized to *M. bovis *BCG or environmental mycobacteria [131]. The rESAT-6 obtained from *E. coli *is also biologically active and was successfully used as a skin test reagent for the diagnosis of tuberculosis infection in humans in phase I clinical trials [132,133]. The sensitivity of rESAT-6 has been enhanced further by combining it with CFP-10 and the ESAT-6/CFP-10 fusion protein was found to be as sensitive as PPD in predicting disease in *M. tuberculosis*-infected guinea pigs [134]. It is expected that rESAT-6/CFP-10 fusion protein could probably replace PPD as skin test reagent for identifying individuals with LTBI.

Other cell mediated immunity-based assays have also been developed. The *in vitro *T cell-based interferon-gamma (IFN-γ) release assays (IGRAs) were developed based on the principle that T cells of individuals sensitized with *M. tuberculosis *antigens produce high levels of IFN-γ in response to a reencounter with these antigens [135]. Initially IGRAs used PPD as the stimulating antigen, however, it was subsequently replaced by two *M. tuberculosis*-specific T cell antigens; ESAT-6 and CFP-10 and the assays were found to be sensitive and specific for detection of active pulmonary/extrapulmonary TB as well as latent infection [136-140].

Two commercial IGRAs, whole blood, ELISA-based QuantiFERON-TB Gold (Cellestis Ltd., Carnegie, Australia) and peripheral blood mononuclear cell (PBMC) and enzyme-linked immunospot (ELISPOT) technology-based T-SPOT.TB (Oxford Immunotec, Oxford, UK) tests were subsequently developed and approved by Food and Drug Administration (FDA) for detecting latent infection. The first-generation QuantiFERON-TB Gold test was based on stimulation of T lymphocytes with PPD and measurement of IFN-γ production [141]. The enhanced QuantiFERON-TB Gold assay subsequently used ESAT-6 and CFP-10 proteins as stimulating antigens. The first-generation T-SPOT.TB used ESAT-6 and CFP-10 proteins as stimulating antigens and detected T-cells themselves [138]. These commercial tests have undergone further improvement since their inception. The newer version of the QuantiFERON-TB Gold assay is called QuantiFERON-TB-Gold-In-Tube (QFT-G-IT) (Cellestis Ltd., Carnegie, Australia) that uses ESAT-6 and CFP-10 and TB7.7 (corresponding to Rv2654 [1]) peptides as antigens. The newer version of T-SPOT.TB also uses peptides of ESAT-6 and CFP-10 instead of whole ESAT-6 and CFP-10 proteins as antigens (Oxford Immunotec, Oxford, UK).

The performance of both QFT-G-IT and T-SPOT.TB tests have been evaluated extensively with/without head-to-head comparison with TST and several systematic reviews are available for their performance in different settings [123-126,142-144]. Similar to TST, a major limitation of both IGRAs is their inability to distinguish LTBI from active TB disease. This may be particularly important in high TB incidence countries in which latent infection is widespread and reinfection happens frequently and in immunocompromised individuals (such HIV-seropositive subjects) and children due to subclinical disease presentation [123,124,126]. However, IGRAs have better specificity (higher that TST) as they are not affected by prior BCG vaccination since the antigens used in these assays are not present in *M. bovis *BCG and cross reactivity with environmental mycobacteria is less likely [123-125]. Furthermore, based on limited data in immunocompromised individuals, the sensitivity of IGRAs, particularly for T-SPOT.TB, is also higher than TST [124]. However, the clinical performance of these tests has been variable in different settings around the globe due to differences in spectrum and severity of TB cases and proportion of HIV-coinfected individuals included in various studies [123,126].

In low TB incidence countries, screening for LTBI aims to identify individuals at higher risk of progression from latent infection to active TB disease. These include all recently infected individuals (close contacts of active pulmonary TB index case), recent immigrants from high TB incidence countries and persons with suppressed (such as HIV coinfected) or immature (such as very young children) cellular immune systems [123,126,142]. Previous data on natural history of TB suggest that after exposure to *M. tuberculosis*, 5-10% of infected individuals develop active TB disease within the first 2 years of initial infection [109,113]. In people with a robust immune system, another 5-10% individuals develop active disease during the remainder of their lives while in immunocompromised individuals, the risk is much higher [123,124]. Thus, diagnosis and treatment of LTBI will be most effective if it is specifically directed to those individuals with the highest risk of progression from LTBI to active disease such as recently exposed individuals, young children and HIV-infected and other immunocompromised subjects.

The current cumulative evidence (summarized in several reviews and meta-analyses) [123-126,142-144] suggest that the performance of the two (ELISA-based and ELISPOT-based) formats of IGRAs are nearly comparable in predicting development of active disease in immunocompetent individuals. However, the agreement between IGRAs and TST is generally poor due to false-positive TST results in BCG vaccinated subjects. The clinical relevance of a positive TST result is usually poor (i.e. unable to predict which patients will develop active TB disease in the near future) and sensitivity as well as specificity are influenced by the different cut-off values used in different settings. However, the value of negative TST result in predicting no further development of active disease in human subjects presumably exposed to *M. tuberculosis *is fairly high (negative predictive value). On the other hand, the predictive value of positive IGRA results for the development of active TB is usually better than that of TST while the predictive value of a negative result is very high in immunocompetent individuals, particularly if the TST is also negative [123-126]. The TST is often negative in immunocompromised individuals and its performance is also influenced by the immunosuppressing conditions while the sensitivity of IGRAs is generally better than TST and the experimental conditions (particularly in T-SPOT.TB assay) can be easily adjusted for testing immunocompromised individuals [124,142].

A major problem associated with IGRAs is the occurrence of indeterminate results that seem to arise mostly due to cellular immune suppression and occur more frequently with the ELISA-based method than with ELISPOT test or discordant results if both, TST and a blood test are performed [123,124]. This is further compounded by the differences that exist in the manner in which these tests are applied for the detection of latently infected individuals in different settings. In the United States and few other countries, national guidelines advocate up-front use of a blood test (IGRA) as a direct replacement for TST in all groups of subjects [145]. Due to higher sensitivity of IGRAs, it is likely that some individuals who are positive for a blood test but who may have been TST negative (if the test was performed) are unnecessarily treated. On the contrary, in the United Kingdom and other European countries, initial screening is performed with TST except in individuals in whom TST is unreliable (young children, HIV-seropositive and other immunosuppressed individuals) [124,146]. For the latter grouping and for TST-positive individuals at higher risk of developing active disease, a blood test is recommended for confirmation of a presumed infection. Thus, it is also probable that a TST-negative subject who may have been IGRA positive will not be identified as having LTBI and will, therefore, not receive treatment. Consequently this apoproach, though supposedly more economical, may result in undertreatment of some individuals with LTBI [123,124]. A discordant result (TST negative but IGRA positive) in an immunocompetent individual should be repeated after 3 months and should be treated for LTBI if IGRA still remains positive (a negative IGRA on repeat testing may signify a transient *M. tuberculosis *infection that was quickly cleared) [124]. However, a similar result in an immunocompromised individual should be carefully evaluated as in this setting, any positive result may be significant.

Although both, TST and IGRAs cannot distinguish between LTBI and active TB disease in immunocompetent adults [123,126], however, in high-risk individuals with immunosuppressive conditions and children, IGRAs may help in the investigation of active disease as adjunctive diagnostic tests, particularly if specimens (such as bronchoalveolar lavage, cerebrospinal fluid) from the suspected site of infection rather than blood is used for the diagnostic assay [147-149]. While the results of IGRAs exhibit better correlation with surrogate measures of exposure to *M. tuberculosis *in low TB incidence countries, however, their performance is generally sub-optimal in countries with a high TB incidence [123-126,143,144,150]. Application of targeted tuberculin skin testing and IGRAs to identify latently infected individuals and their treatment for LTBI has greatly helped in lowering the incidence of TB in rich, advanced countries [128,138,140,144,151]. Previous studies have shown that majority of active disease cases in low or low-intermediate incidence countries in immigrants/expatriates originating from TB endemic countries occur as a result of reactivation of previously acquired infection mostly within two years of their migration [6,9,113,114,140]. Some other low-intermediate TB incidence countries which contain large expatriate populations originating from TB endemic countries are also evolving similar strategies for controlling TB [152-157].

Another variation of conventional cell mediated immunity-based assays (IGRAs) has also been developed by using flow cytometry [158]. Although flow cytometric approach uses smaller blood volume (<1 ml), the assay will have limited utility in much of the developing world due to the high cost of flow cytometers and the need for technically experienced personnel. The detection of significant levels of antibodies to some *M. tuberculosis*-specific proteins has also been noted in contacts of TB patients (latently infected individuals) as well as in patients with active TB disease but not in healthy subjects [159-162]. However, antibody-based methods are only experimental and are not used in clinical practice for the detection of LTBI.

## Treatment of latent *M. tuberculosis *infection

Tracing contacts of infectious pulmonary TB cases (sputum smear-positive) for exposure to tubercle bacilli leading to latent *M. tuberculosis *infection (LTBI) and treatment of latently-infected individuals at high risk of progressing from latent infection to active disease has proven extremely effective in the control of TB in the United States and other low TB-burden countries [128,151,163]. Treatment of LTBI in infected persons substantially reduces the likelihood of activation of dormant infection and subsequent development of active TB disease (Figure 1). The American Thoracic Society (ATS) and Centers for Disease Control and Prevention (CDC) issued guidelines in 2000 for the treatment of LTBI which were also endorsed by the Infectious Diseases Society of America and American Academy of Pediatrics [128]. An update to these guidelines was published in 2005 that also included recommendations for pediatric subjects [164]. The treatment options currently available for LTBI are summarized in Table 2.

**Table 2:** Currently available drug regimens for the treatment of latent tuberculosis infection

Drug(s)	Adult maximum dose(s) (mg)	Duration of treatment	Drug intake	Frequency	Comments
INH	300	9 months	Self administered	Daily	Preferred regimen by CDC

INH	900	9 months	Under DOT	2/Wk	Alternative regimen

INH	300	6 months	Self administered	Daily	For HIV seronegative only

INH	900	6 months	Under DOT	2/Wk	For HIV seronegative only

INH	300	12 months	Self administered	Daily	Preferred regimen by IUAT

RMP	600	4 months	Self administered	Daily	For LTBI with INH^r ^strain in HIV seronegative subjects

INH + RMP	300 + 600	3 months	Self administered	Daily	Good alternative option

RMP + PZA	600 + 2000	2 months	Self administered	Daily	Higher risk of hepatotoxicity

RMP + PZA	600 + 2500	2 months	Under DOT	2/Wk	Higerh risk of hepatotoxicity

INH + RPE	900 + 900	3 months	Under DOT	1/Wk	Promising option

INH, isoniazid; RMP, rifampicin; PZA, pyrazinamide; RPE, rifapentine; DOT, directly observed treatment; 2/Wk, twice weekly; 1/Wk, once weekly; CDC, Center for Disease Control and Prevention; HIV, human immunodeficiency virus; IUAT, international Union Against Tuberculosis; LTBI, latent tuberculosis infection

The standard regimen for the treatment of LTBI in United States and Canada is daily self-administered therapy with isoniazid (INH) for nine months based on clinical trial data but the duration of treatment can be reduced to 6 months for adults seronegative for HIV-infection [128,164]. The International Union Against Tuberculosis (IUAT) recommends daily therapy with INH for 12 months as it is more effective than the 6-month course (75% vs. 65%) [165]. The preferred duration of treatment for most patients with LTBI in the United States and European countries is 9 months since clinical trial data showed that the efficacy of 6-month regimen is reduced to 60% while 12-month regimen is advocated for individuals at higher risk of developing active disease [123,166]. According to the CDC guidelines, the frequency can also be reduced from daily therapy to twice weekly therapy with increased dosage of INH, however, the twice weekly regimen must be given as directly observed treatment (DOT) [164]. Inclusion of DOT adds a substantial additional expense to the treatment strategies. The efficacy of INH treatment in preventing active TB exceeds 90% among persons who complete treatment [165]. However, the overall effectiveness of these regimens is severely limited as the completion rates in clinical settings have been rather low, ranging from 30% to 64% only [167-169]. Completion rates in other settings have been even lower [170]. Although INH is tolerated fairly well by most of the individuals, there is a risk of hepatic toxicity in selected populations. Studies have shown that 10% to 22% of participants taking INH for LTBI have at least one episode of elevated serum transaminase levels. Although the rates of clinically significant hepatitis were much lower (< 2%), the risk and severity increased with age and concomitant alcohol consumption [171-173]. INH can also cause peripheral neuropathy but the risk can be lowered by concomitant use of pyridoxine (vitamin B6) [174]. Poor adherence due to the long duration of treatment and concerns for hepatotoxicity in selected patient populations resulted in development of shorter and more effective treatment options for LTBI [128,164].

The ATS and CDC guidelines also included 4 months of rifampicin (RMP) alone or 2 months of RMP and pyrazinamide (PZA) as acceptable alternatives for the treatment of LTBI [128]. The RMP alone is recommended for persons intolerant to INH, close contacts of TB cases in which the isolate of *M. tuberculosis *is resistant to INH or INH resistance is suspected due to the origin of foreign-born persons from countries where INH resistance rates are high [128,175,176]. There are several advantages with 4 month daily therapy with RMP such as lower cost, higher adherence to treatment and fewer adverse reactions including hepatotoxicity [151,169,177-180]. However, treatment with RMP alone is not recommended for HIV-seropositive persons on concomitant anti-retroviral therapy as this may lead to the development of acquired rifamycin resistance [164,181,182]. Furthermore, active disease in an HIV-infected individual should be ruled out first since monodrug therapy in an undiagnosed active TB disease case may also lead to RMP resistance. However, active TB disease is more difficult to exclude in HIV-infected individuals as they are less likely to have typical features of pulmonary TB and extrapulmonary TB occurs more frequently [6,183,184]. The regimen of RMP alone is also not suitable for patients with other underlying conditions such as diabetes [185,186].

Treatment of LTBI with RMP + PZA for 4 months is another alternative choice that was advocated by ATS and CDC guidelines in 2000 [128]. Although initial studies with 2 months of RMP + PZA in HIV-infected persons were reported to be as effective and safe as INH treatment [187,188], several cases of severe liver injury and/or death were reported subsequently with the RMP + PZA regimen resulting in revision of ATS/CDC recommendations in 2003 [189]. The revised guidelines advocated that 2 months of RMP + PZA regimen should not generally be offered to HIV-seronegative or HIV-seropositive individuals [163,164]. A meta-analysis involving six clinical trials comparing the effectiveness of 2 months of RMP + PZA with 6 or 12 months of INH treatment showed that RMP + PZA regimen was associated with increased risk of hepatotoxicity in HIV seronegative persons while the results for HIV-infected persons were inconclusive [190]. However, when the results of 2 months of RMP + PZA were compared with 6 months of INH treatment without supplementation with pyridoxine in HIV-infected persons, the data showed no significant differences in hepatotoxicity in the two sub-groups. The results of some studies suggest that 2 months of RMP + PZA regimen may also be considered when other regimens are unsuitable and monitoring of liver function tests is feasible [191,192].

Other options that have been tested or are under evaluation for the treatment of LTBI include 3 months of INH + RMP given daily or twice weekly under DOT and 3 months of INH + rifapentin (RPE) given once weekly. The 3 months of INH + RMP regimen has been tested mostly in the United Kingdom. A meta-analysis of five studies carried out in both HIV-infected and HIV-seronegative individuals as well as two subsequent studies have shown that the 3 month of INH + RMP treatment is well tolerated and is as effective and safe as 6 to 12 months of INH treatment alone [193-195]. The longer half life of RPE, approved by U. S. Food and Drug Administration (FDA) in 1998 for the treatment of TB, has allowed once weekly dosing of INH + RPE for the treatment of LTBI [196]. One small study comparing once-weekly INH + RPE for 3 months with daily RMP + PZA for 2 months reported fewer discontinuation of treatment due to hepatotoxicity in the INH + RPE arm compared to the RMP + PZA arm even though the risk of developing active TB was nearly same in both the groups [197]. A large multi-center study is currently being conducted by the Tuberculosis Trials Consortium of the CDC to determine the efficacy of once weekly dosing of INH + RPE in preventing active disease among high-risk individuals with LTBI. However, the cost of once weekly regimen of INH + RPE is an important issue since RPE is currently more expensive than RMP.

## Future prospects

A major concern that has arisen recently is the threat of latent infection in a person exposed to a source case infected with multidrug-resistant strain of *M. tuberculosis *(MDR-TB). As nearly 440 000 cases of MDR-TB corresponding to nearly 5% of all incident TB cases occurred in 2008 [5], this concern is likely to attract greater attention in the near future. Only scant information is available in this setting as there have been no randomized controlled trials to assess the effectiveness of specific regimens [198]. A 6 to 12 month regimen of a fluoroquinolone + pyrazinamide or ethambutol + pyrazinamide is recommended by CDC. However, the effectiveness and optimal duration of these regimens is largely unknown as they are very poorly tolerated [199]. The newer drugs that are in different stages of development may offer better alternatives for the treatment of both, active TB disease as well as LTBI.

The new generation fluoroquinolones such as moxifloxacin have excellent (bactericidal) activity against *M. tuberculosis *and may be more effective in the treatment of LTBI than older drugs of the same class [200,201]. In experimental animal model of latent infection, the once weekly regimen of rifapentine + moxifloxacin for 3 months was found to be as effective as daily therapy with isoniazid for 9 months [202]. The PA-824, a nitroimidazo-oxazine, is another promising compound that is active against MDR-TB strains and is also active against non-replicating persistent bacteria, making it an ideal drug candidate for the treatment of LTBI. The treatment regimen containing PA-824, moxifloxacin, and pyrazinamide was highly effective in murine model of tuberculosis [203]. The OPC-67683, a nitroimidazo-oxazone, is another promising new compound that shows promising results against tuberculosis in mice [204]. A diarylquinoline (R207910 also known as TMC207) has shown more potent early bactericidal activity than INH during early phase of infection and higher bactericidal activity late in infection than RMP alone and thus may provide another option for the treatment of LTBI [205,206]. Another promising drug is SQ109 (1,2-ethylenediamine) that is structurally related to ethambutol but is more potent [207,208]. It is expected that some of these new drugs will provide additional options for the treatment of LTBI in the near future.

Another approach that is actively being pursued for controlling development of active disease in persons with LTBI is development of novel vaccines that may prevent TB disease reactivation by efficiently containing the pathogen in a latent state in infected individuals [209-211]. More than 10 vaccine candidates have entered clinical trials in the past few years [209]. Two of these vaccine candidates are recombinant *M. bovis *BCG constructs designed to improve the antigenicity and/or immunogenicity of the current BCG vaccine [212,213]. Another seven subunit vaccines are being tested in clinical trials and are being used as booster vaccines designed to reorient the immune response after priming with recombinant BCG vaccines. Three of the subunit vaccines are incorporated in viral carriers while the other four subunit vaccines are being delivered through adjuvant formulations [209,214-216]. The recombinant BCG and booster subunit vaccines are designed to be given prior to *M. tuberculosis *infection to sustain latent infection and either prevent or delay the reactivation of latent infection by inducing a memory T cell response that resists exhaustion and suppression [209]. Other vaccine candidates under development include further modifications such as inclusion of dormancy-regulated genes to improve the efficacy of BCG replacement vaccine candidates for post-exposure vaccination of latently infected individuals (Figure 1) [101,209]. A drawback of the above vaccines is that they prevent or delay the reactivation of dormant infection but do not eradicate the pathogen. However, attempts are now underway to combine the antigens of metabolically active (such as secreted proteins) and dormant (such as dormancy-regulated genes) state of *M. tuberculosis *in both, the recombinant BCG and subunit booster vaccines to achieve sterile eradication of the pathogen [209].

## Conclusion

Infection with *M. tuberculosis *begins with the phagocytosis of tubercle bacilli by antigen-presenting cells in human lung alveoli. This sets in motion a complex infection process by the pathogen and a potentially protective immune response by the host. *M. tuberculosis *has devoted a large part of its genome towards functions that allow it to successfully establish progressive or latent infection in majority of infected individuals. The failure of immune-mediated clearance is due to multiple strategies adopted by *M. tuberculosis *that blunt the microbicidal mechanisms of infected immune cells and formation of distinct granulomatous lesions that differ in their ability to suppress or support the persistence of viable *M. tuberculosis *(LTBI). A positive tuberculin skin test or T cell-based interferon-γ release assay in a person with no overt signs of active disease indicates LTBI and requires treatment of individuals particularly those at the highest risk of progression from LTBI to active disease such as recently exposed individuals, young children and HIV-infected and other immunocompromised subjects. Standard treatment regimen for LTBI is daily therapy with isoniazid for nine months. New drugs/drug combinations as well as novel vaccine approaches are being developed for eradication of latent infection in exposed individuals. Identification and treatment of latently infected individuals has greatly helped in control of TB in rich, advanced countries and similar approaches hold great promise for other countries with low-intermediate rates of TB incidence.

## Competing interests

The author declares that they have no competing interests.
